# Bone marrow adipoq^+^ cell population controls bone mass via sclerostin in mice

**DOI:** 10.1038/s41392-023-01461-0

**Published:** 2023-07-10

**Authors:** Huanqing Gao, Yiming Zhong, Sixiong Lin, Qinnan Yan, Xuenong Zou, Guozhi Xiao

**Affiliations:** 1 Department of Biochemistry, School of Medicine, Guangdong Provincial Key Laboratory of Cell Microenvironment and Disease Research, Shenzhen Key Laboratory of Cell Microenvironment, Southern University of Science and Technology, Shenzhen, 518055 China; 2 State Key Laboratory of Genetic Engineering and School of Life Sciences, Fudan University, Shanghai, 200438 China; 3 Guangdong Provincial Key Laboratory of Orthopedics and Traumatology, Department of Spinal Surgery, The First Affiliated Hospital of Sun Yat-sen University, Guangzhou, 510080 China

**Keywords:** Bone development, Bone remodelling

**Dear editor**,

The comorbidity of obesity and osteoporosis illustrates the communication and coordination of adipose and bone tissues. Leptin and adiponectin derived from adipocytes regulate osteoblast formation and function to impact bone mass through direct and indirect mechanisms.^1^ It is known that bone marrow adipocytes (BMA) can control bone mass by modulating the bone morphogenetic protein (BMP) and other signaling pathways. BMAs can secret soluble factors, which impact osteoblasts, osteoclasts, and osteocytes.^2^ Sclerostin is a potent inhibitor of bone acquisition that antagonizes Wnt/β-catenin signaling. Deleting sclerostin was recently reported to protect against cardiovascular disease.^3^ Furthermore, neutralizing monoclonal antibodies against sclerostin increase bone mass and are utilized to treat osteoporosis. Previous studies revealed that global ablation of sclerostin increased both trabecular and cortical bone mass^4^ and that sclerostin produced by the osteocytes located in the bone matrix negatively regulated bone mass in mice.^5^ However, it is not known whether sclerostin derived from other cell types also contributes to bone formation.

Hence, we have explored the contribution of adiponectin-expressing cells-derived sclerostin in control of bone mass by ablating of *Sost* gene, which encodes sclerostin, using the *Adipoq-Cre* that mainly targets adipose lineage cells. We found that mice lacking sclerostin in adiponectin-expressing cells (*Sost*^*Adipoq*^) had similar body weight, fat mass, and organs weight compared to their control littermates (Fig. 1a and Supplementary Fig. 1a–e). The adipocyte size of peripheral adipose tissue was not markedly impacted by *Sost* deletion (Fig. 1b). Results from the glucose tolerance test and insulin tolerance test showed that *Sost* ablation in adipoq^+^ cells did not affect the ability to clear blood glucose (Supplementary Fig. 2a, b) and insulin sensitivity (Supplementary Fig. 2c, d). These results demonstrate that sclerostin loss in adipocytes has no marked effects on peripheral fat mass and glucose metabolism.

**Fig. 1:**
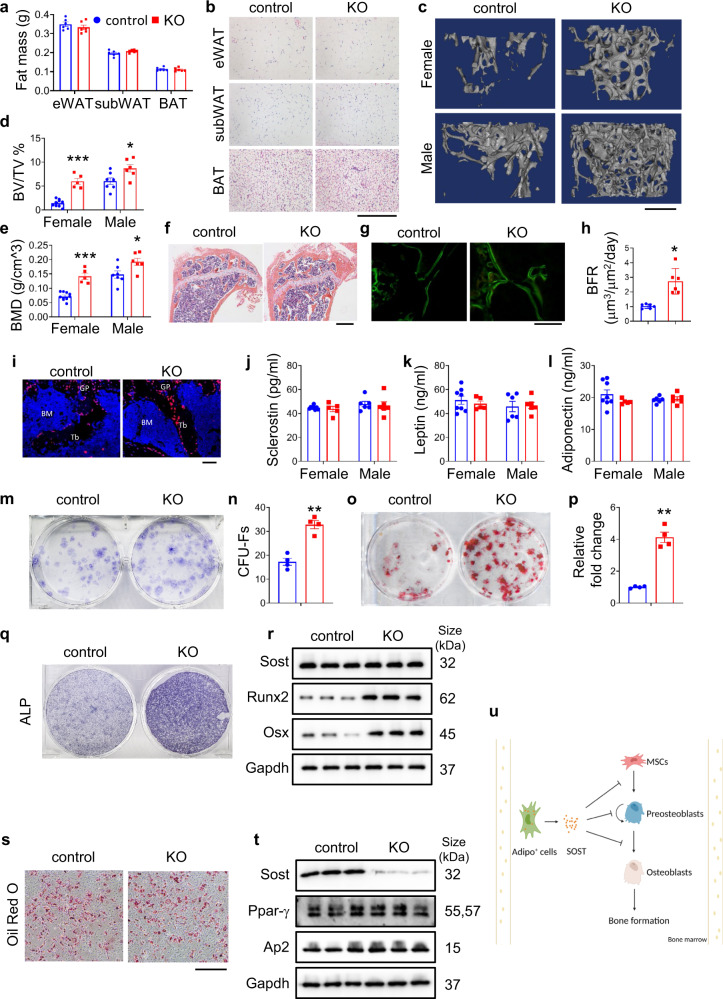
**a** Fat mass of control and KO male mice at the age of 5 months. *N* = 6 for each group. **b** H&E staining of epididymal White Adipose Tissue (eWAT), subcutaneous White Adipose Tissue (subWAT), and Brown Adipose Tissue (BAT) of control and KO female mice fed normal chow diet for 5 months. Scale bar, 50 μm. **c** Three-dimensional (3-D) reconstruction from micro-computerized tomography (μCT) scans of distal femurs from 5-month-old control and KO mice. Scale bar, 500 μm. Quantification of bone volume/tissue volume (**d**), bone mineral density (**e**). *N* = 10 for female control; *N* = 5 for female KO; *N* = 7 for male control; *N* = 6 for male KO. **f** H&E staining of tibial from 5-month-old female mice. Scale bar, 100 μm. **g** Calcein double labeling staining. Representative images of 5-month-old control and KO tibial sections. Scale bar, 50 μm. **h** Quantification of the bone formation rate of trabecular bone. **i** IF staining of osterix. Scale bar, 50 μm. Serum levels of sclerostin (**j**), leptin (**k**) and adiponectin (**l**) from 5-month-old control and KO mice. *N* = 8 for female control; *N* = 5 for female KO; *N* = 6 for male each group. **m, n** Colony forming unit-fibroblast (CFU-F) assays and quantification. **o**, **p** Colony forming unit-osteoblast (CFU-OB) assays and quantification. Primary BMSC was obtained from 5-month-old control and KO female mice and cultured with osteoblast differentiation medium for 7 days. Cells were used for ALP staining (**q**) and Western blot analysis with the indicated antibodies (**r**). *N* = 3 biologically independent experiments. Primary BMSCs obtained from 5-month-old control and KO female mice were cultured with adipogenic medium for 7 days. Cells were used for Oil Red O staining (**s**) and Western blot analysis with the indicated antibodies (**t**). Scale bar, 200 μm. *N* = 3 biologically independent experiments. **u** A schematic illustrating how an adipoq^+^ cell population controls bone mass by producing sclerostin in the bone marrow microenvironment. Figure created using BioRender.com. *P < 0.05, **P < 0.01, ***P < 0.001 vs. controls. Results are expressed as mean ± SEM

Results from μCT analyses of skeleton revealed that *Sost*^*Adipoq*^ mice did not show marked alteration in bone mass at 1 month of age (Supplementary Fig. 3a-g). However, at 3 months of age, *Sost*^*Adipoq*^ mice showed an increased bone mass (Supplementary Fig. 4a–f). Moreover, at the age of 5 months, bone mass of *Sost*^*Adipoq*^ mice was significantly increased compared to control littermates, especially in female group (Fig. 1c). *Sost* deletion significantly increased the femoral bone volume/total volume, bone mineral density, trabecular number, and trabecular thickness and decreased the trabecular separation without impacting the cortical thickness (Ct.Th) (Fig. 1d, e and Supplementary Fig. 5a–d). Note: The Ct.Th was reported to be significantly increased in the global *Sost* knockout mice.^4^ The skull size and shape were similar between the two groups (Supplementary Fig. 6a). Furthermore, the spine bone mass was not affected by *Sost* deletion (Supplementary Fig. 6b–g). Hematoxylin and eosin (H&E) staining of the tibial sections revealed more trabecular bone in *Sost*^*Adipoq*^ mice than in control littermates (Fig. 1f). We performed the calcein double-labeling experiments and found that the tibial bone formation was significantly accelerated in *Sost*^*Adipoq*^ mice, as demonstrated by significant increases in the mineral apposition rate, mineralizing surface per bone surface and bone formation rate in *Sost*^*Adipoq*^ versus control mice (Fig. 1g, h and Supplementary Fig. 7a, b). The increased bone mass in *Sost*^*Adipoq*^ mice could be due to increased bone formation and/or decreased bone resorption. Thus, we further determined the effect of sclerostin loss on bone resorption. Tartrate-resistant acid phosphatase staining of bone sections indicated that osteoclast formation in *Sost*^*Adipoq*^ mice was comparable to that in control littermates (Supplementary Fig. 8a, b). We further measured the serum levels of collagen type I cross-linked C-telopeptide, a biomarker for bone resorption, and observed no significant difference between the two groups (Supplementary Fig. 8c). Osterix immunofluorescence staining revealed more osteoblasts around the trabeculae in *Sost*^*Adipoq*^ mice than in control littermates (Fig. 1i). We next examined the effect of sclerostin loss on bone mass in mice with ovariectomy (OVX). We found that *Sost* deletion ameliorated to certain extent the osteoporotic phenotypes induced by estrogen deficiency (Supplementary Fig. 9a–g).

The serum level of sclerostin protein was not significantly different between the two genotypes (Fig. 1j). Furthermore, *Sost* deletion did not change the serum levels of leptin and adiponectin, which are known to impact bone mass (Fig. 1k, l). Collectively, these results suggest the notion that it is unlikely that the high bone mass in *Sost*^*Adipoq*^ mice is due to systemic sclerostin loss. For this reason, we next analyzed the bone marrow tissues of both genotypes. Consistent with results from peripheral fat mass analyses, perilipin staining in bone marrow was comparable, indicating that *Sost* deletion does not affect the adipocyte number and size in bone marrow tissue (Supplementary Fig. 10). We found that *Sost* inactivation promoted the formation of the bone morrow-derived colony-forming units-fibroblast (CFU-F) (Fig. 1m, n) and colony-forming units-osteoblast (CFU-OB) (Fig. 1o, p). IF staining showed that the expression level of active-β-catenin protein was increased in KO bone compared to that in control bone (Supplementary Fig. 11). The expression levels of osteogenic marker proteins Runx2 and osterix (Osx) and alkaline phosphatase (Alp) activity, an early marker of osteogenesis, were dramatically increased in primary bone marrow stromal cell (BMSC) cultures from *Sost*^*Adipoq*^ mice compared to those from control mice (Fig. 1q, r). Notably, the expression levels of adipogenic factors Ppar-γ and AP2 and the adipogenic differentiation capacity of the BMSC cultures, as determined by Oil Red O staining, were not affected by Sost loss (Fig. 1s, t). Thus, for the first time to our knowledge, we establish that the bone marrow adipoq^+^ cell population plays an important role in promoting BMSC osteoblast differentiation and bone formation (Fig. 1u). This unique cell population in the bone marrow may be a useful target for osteoporosis treatment.

## Supplementary information

supplement material

## Data Availability

Data are available upon reasonable request.
