# Supplementary material for: Bone marrow adipoq+ cell population controls bone mass via sclerostin in mice
Source: Signal Transduct Target Ther. 2023 Jul 10;8:265. doi: 10.1038/s41392-023-01461-0 (PMC10330180; doi:10.1038/s41392-023-01461-0)
Supplement: Supplementary file 1 — supplement material [file 41392_2023_1461_MOESM1_ESM.docx]

Supplementary Materials for

**Bone marrow adipoq^+^ cell population controls bone mass via sclerostin in mice**

Huanqing Gao^1,2#^*, Yiming Zhong^1#^, Sixiong Lin^1,3#^, Qinnan Yan^1^, Xuenong Zou^3^, Guozhi Xiao^1^*

^1^Department of Biochemistry, School of Medicine, Guangdong Provincial Key Laboratory of Cell Microenvironment and Disease Research, Shenzhen Key Laboratory of Cell Microenvironment, Southern University of Science and Technology, Shenzhen 518055, China.

^2^State Key Laboratory of Genetic Engineering and School of Life Sciences, Fudan University, Shanghai 200438, China.

^3^Guangdong Provincial Key Laboratory of Orthopedics and Traumatology, Department of Spinal Surgery, The First Afﬁliated Hospital of Sun Yat-sen University, Guangzhou 510080, China.

^#^These authors contribute equally to this paper.

*Address correspondence to: GX (email: [xiaogz@sustech.edu.cn](mailto:xiaogz@sustech.edu.cn)) or HG (gaohuanqing@126.com).

**This file includes:**

Materials and Methods

Figures. S1 to S11

Supplementary Table 1

References for supplementary materials

**Materials and Methods**

**Animal study**

*Adipoq-Cre* mice and *Sost^flox^* mice were obtained from The Jackson Laboratory. To obtain KO mice, *Sost^flox/flox^* mice were crossed with *Adipoq-Cre* mice. All mice were kept in temperature-controlled (23 ± 1) °C facilities with a 12 h light-dark cycle and stable humidity (50% ± 10%) conditions. All animal experimentation was approved by the SUSTech Animal Care and Use Committee.

**Micro-computed tomography (Micro CT)**

Bone samples were fixed with 10% formalin. CT scanning was performed using a Bruker Micro CT (SkyScan 1172 Micro-CT, Bruker MicroCT) following the standards of techniques and terminology suggested by the American Society for Bone and Mineral Research. Bone parameters were calculated as previously described ^1, 2^, including the bone mineral density (BMD), trabecular separation (Tb.Sp), bone volume per tissue volume (BV/TV), trabecular number (Tb.N), trabecular thickness (Tb.Th), and cortical thickness (Ct.Th).

**Histology and histomorphometry**

Epididymal WAT was fixed in 10% formalin, dehydrated, embedded in paraffin and stained with hematoxylin and eosin (H&E). For bone histology, mouse tibiae were fixed with 10% formalin for 24 hours, decalcified in 10% EDTA for 3 weeks, embedded in paraffin, and stained by H&E, or tartrate-resistant acid phosphatase (TRAP).

**Calcein labeling**

Mice were intraperitoneally injected with 20 mg/kg calcein (Sigma, cat# C0875) on days 7 and days 2 before sacrifice. The images were obtained using a fluorescence microscope and the MAR (mineral apposition rate), MS/BS (bone formation rate/bone surface), and BFR (bone formation rate) were evaluated as we previously described.

**Glucose tolerance test and insulin tolerance test**

For the glucose tolerance test (GTT), the mice were fasted overnight with unrestricted access to water. The following morning, mice were weighed and blood was collected to measure baseline glucose levels (0 min). The mice were then given an intraperitoneal injection (1.5 g/kg body weight). Tail blood glucose levels were determined at 15, 30, 60, and 120 minutes after injection using a Bayer Contour glucometer. For the insulin tolerance test (ITT), mice were fasted for 6 h with free access to water. The mice were weighed, and blood glucose levels were measured at 0, 15, 30, 60, and 120 minutes after intraperitoneal injection of insulin at a dose of 0.5 U/kg body weight.

**Western blotting**

Western blotting was performed as previously described^3, 4^. In brief, cells were lysed in radioimmunoprecipitation assay (RIPA) buffer containing protease inhibitor and quantified using the BCA protein assay. The cell lysates were resolved on SDS-PAGE gels and transferred to PVDF membranes. Membranes were blocked with 5% skimmed milk for 1 h at room temperature and incubated with primary antibodies 4°C overnight. Membranes were then incubated with HRP conjugated secondary antibodies for 1 h at room temperature, followed by Enhanced chemiluminescence (ECL) detection using Bio-Rad Imaging System. Antibodies information was shown in Supplementary Table 1.

**Immunofluorescent staining**

Immunofluorescent (IF) staining was conducted as we previously described^1, 2^.

**In vitro bone marrow stromal cells culture and differentiation**

Primary bone marrow stromal cells (BMSCs) were isolated from 5-month-old control and KO female mice and cultured as we previously described ^5^. To induce osteoblast differentiation, BMSCs were cultured in osteogenic medium (α-MEM containing 20% FBS and 50 mg/mL ascorbic acid) for 7 days followed by qPCR analysis, western blotting and ALP staining (BCIP/NBT ALP color development kit, Beyotime, cat# C3206). Then, the BMSCs were switched to mineralization-inducing medium (osteogenic medium plus 2.5 mM b-glycerophosphate) for another 7 days for alizarin red S staining using Alizarin Red S Staining Kit (Beyotime, cat# C0148S). To induce adipogenic differentiation, BMSCs were cultured with adipogenesis Kit (Stemcell Technologies, cat# 05507) for 7 days, followed by qPCR, western blotting analysis and Oil red O staining (Sigma, cat# O0625).

**CFU-OB and CFU-F assays**

Primary BMSCs were obtained from 5-month-old femurs and tibias of control and KO female mice and cultured as we previously described ^5^. The CFU-OB and CFU-F assays were performed as we described previously ^6^.

**Serum ELISA**

The whole blood of mice was coagulated at room temperature for 1 h, centrifuged at 2000g for 10 min at 4°C, and the supernatant was taken. Store immediately in the refrigerator at −80°C. Serum levels of CTX-1(Immunodiagnostic Systems Limited, cat# AC-06F1), leptin (R&D, cat# MOB00) and adiponectin (R&D, cat# MRP300) were detected by ELISA kit.

**OVX mouse model**

Females at 4 months of age were anesthetized and ovariectomized as described in previous^7^.

**Statistical analysis**

Data were statistically analyzed using an unpaired two-tailed Student’s *t*-test. The results are representative of at least three biologically independent experiments. Quantitative values are presented as the mean ± SEM. Statistical significance is indicated as **P* < 0.05, ***P* < 0.01, and ****P* < 0.001.


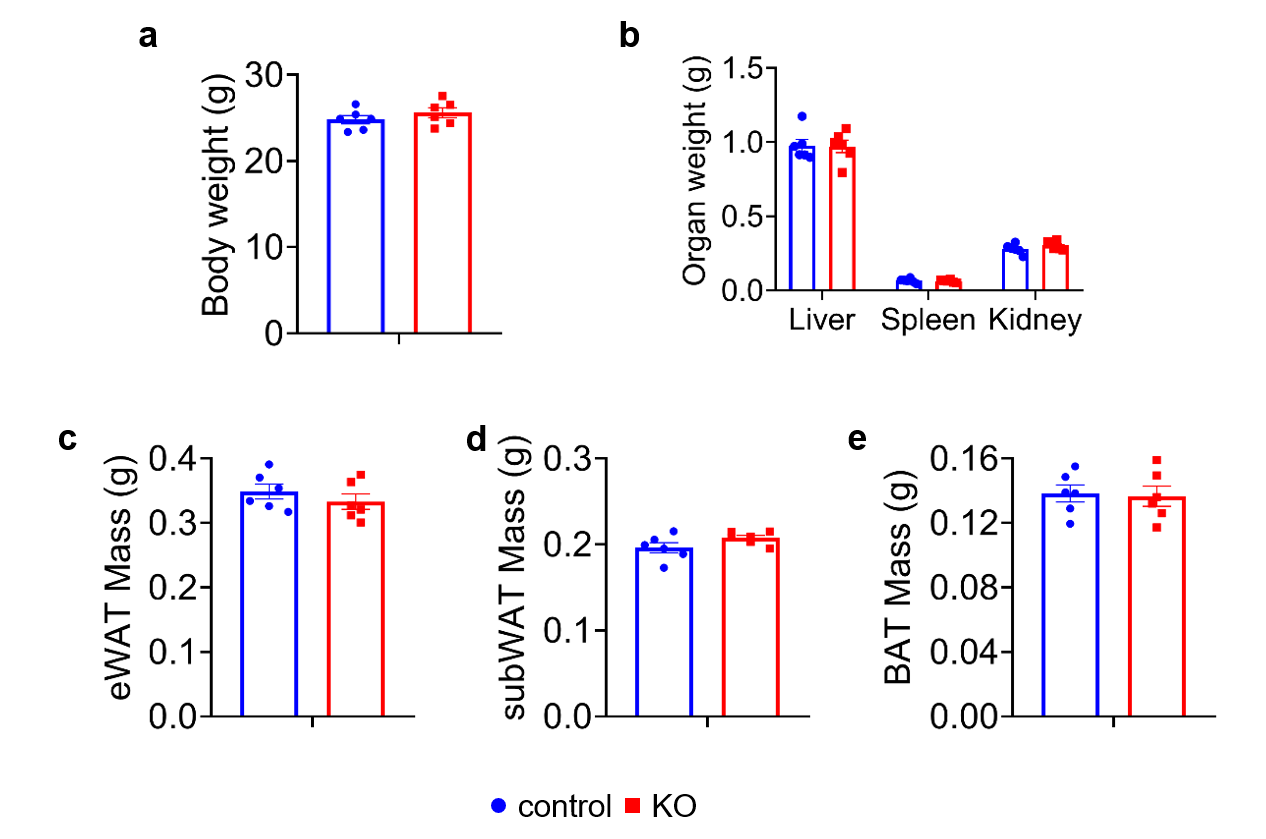


**Supplementary Figure 1. a** Body weight of control and KO male mice fed NCD at the age of 5 month. *N* = 6/group. **b** Organs’ weight. *N* = 6/group. **c-e** Quantification of fat pads including eWAT (**c**), subWAT (**d**), and BAT (**e**) from NCD fed mice. *N* = 6/group. Results are expressed as mean ± SEM.


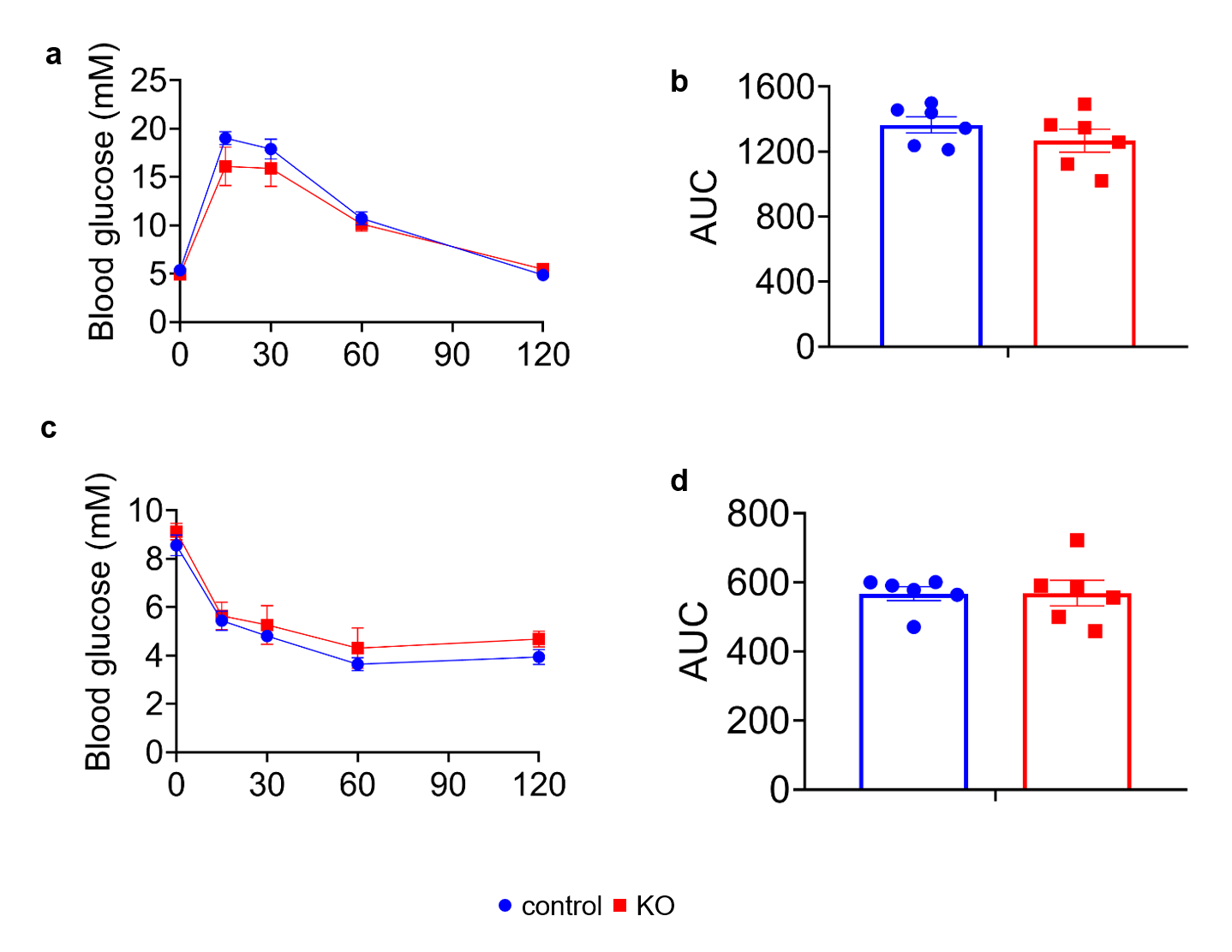


**Supplementary Figure 2. a** Blood glucose concentration during GTT from male mice fed NCD for 5 months. **b** Area under curve (AUC) calculated based on data in (**a**). *N* = 6/group. **c** Blood glucose concentrations during ITT from mice fed NCD for 5 months. **d** AUC calculated based on data in (**c**). *N* = 6/group. Results are showed as mean ± SEM.


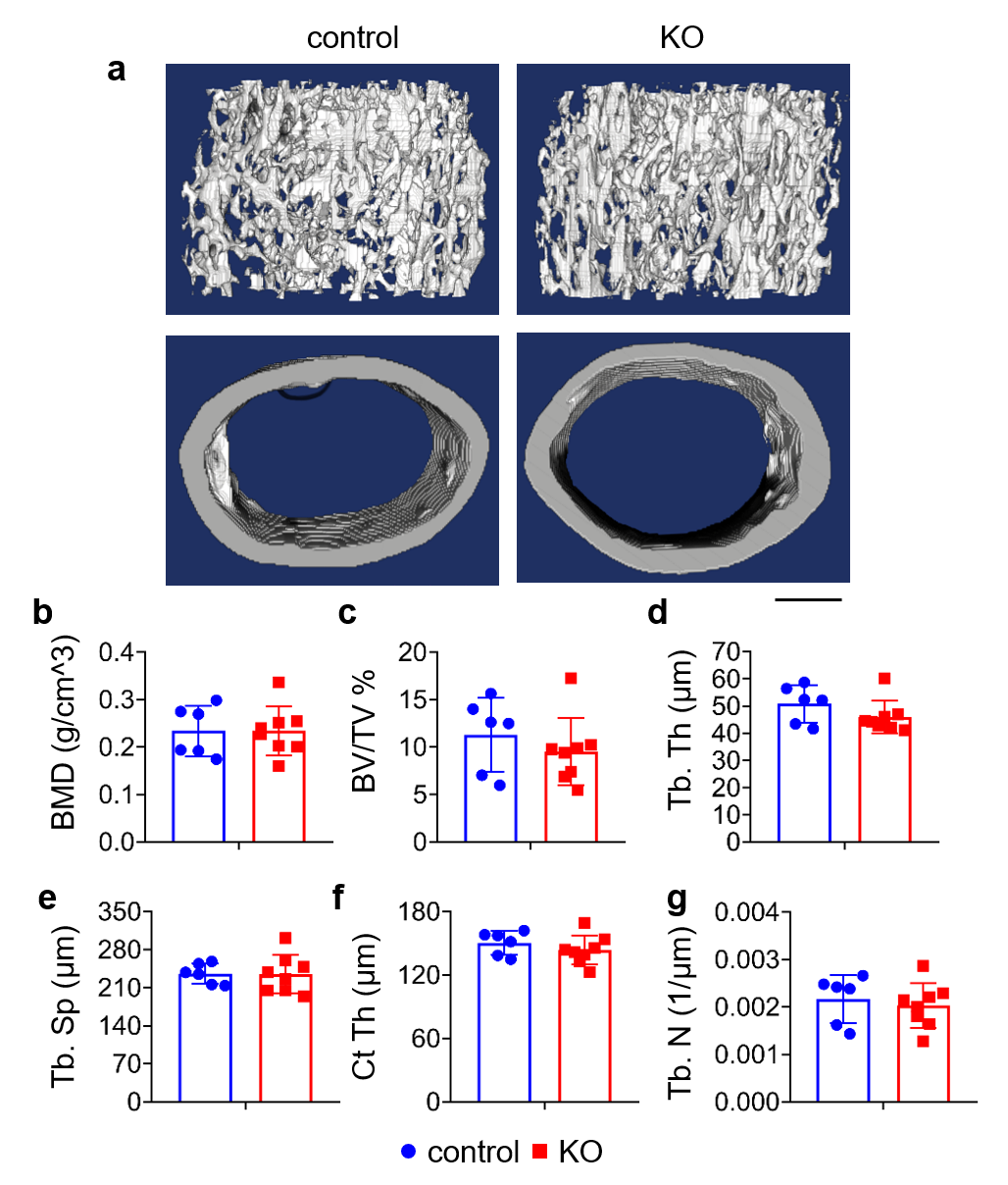


**Supplementary Figure 3. a** Three-dimensional (3-D) reconstruction from micro-computerized tomography (μCT) scans of distal femurs from 1-month-old female control and KO mice with NCD. Scale bar, 500 μm. **b**–**g** Quantitative analyses of bone mineral density (**b**), bone volume/tissue volume (**c**), trabecular thickness (**d**), trabecular separation (**e**), cortical thickness (**f**) and trabecular number (**g**) of distal femurs from **a**. *N* = 6 for control female mice and *N* = 8 for KO female mice. Results are showed as mean ± SEM.


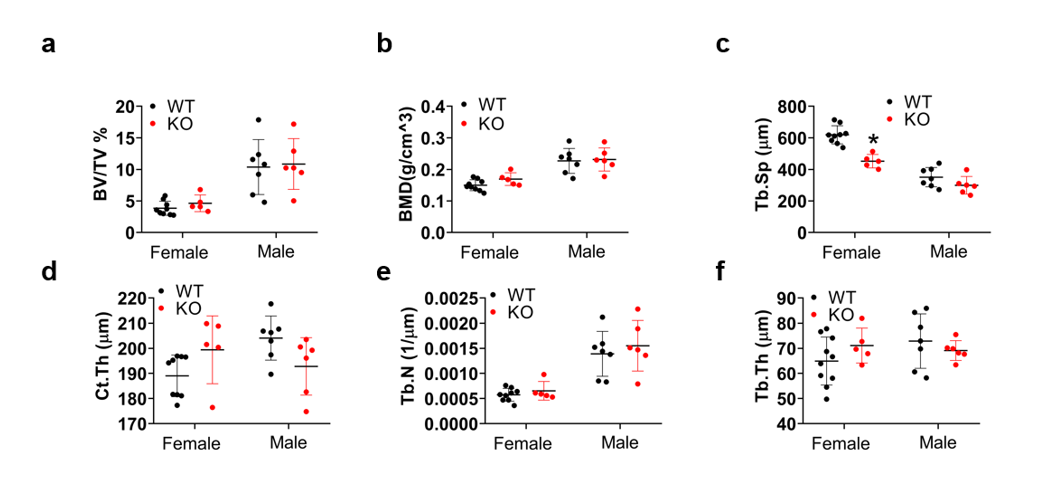


**Supplementary Figure 4.** Quantitative analyses of micro-CT of femur from 3-month-old control and KO mice. **a**. bone volume/tissue volume (BV/TV), **b**. bone mineral density (BMD), **c**. trabecular separation (Tb.Sp), **d**.cortical thickness (Ct.Th), **e**. trabecular number (Tb.N), **f**. trabecular thickness (Tb.Th). *N* = 10 for female control; *N* = 5 for female KO; *N* = 7 for male control; *N* = 6 for male KO. **P* < 0.05 vs. controls.


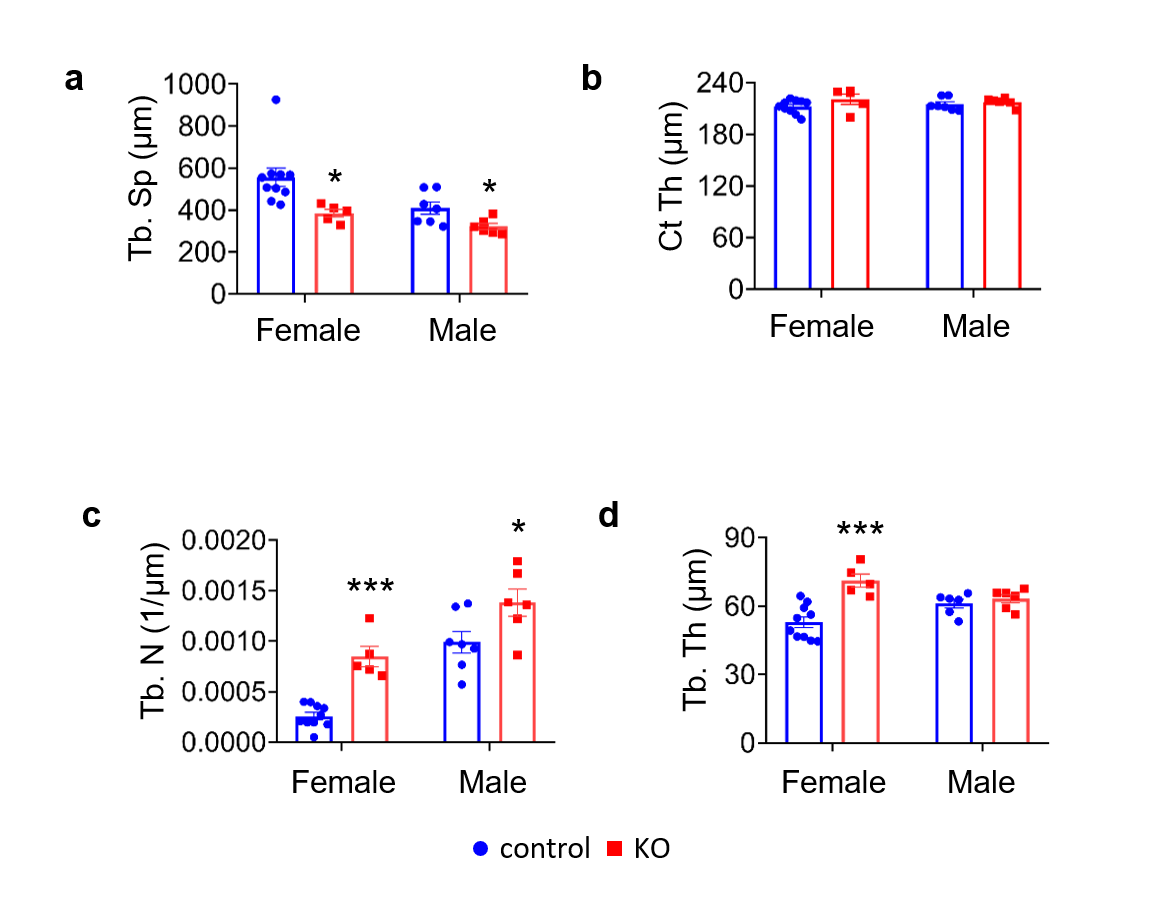


**Supplementary Figure 5. a-d** Quantitative analyses of trabecular separation (**a**), cortical thickness (**b**), trabecular number (**c**) and trabecular thickness (**d**) of distal femurs. *N* = 10 for female control; *N* = 5 for female KO; *N* = 7 for male control; *N* = 6 for male KO. **P* < 0.05, ***P* < 0.01, ****P* < 0.001 vs. controls. Results are showed as mean ± SEM.


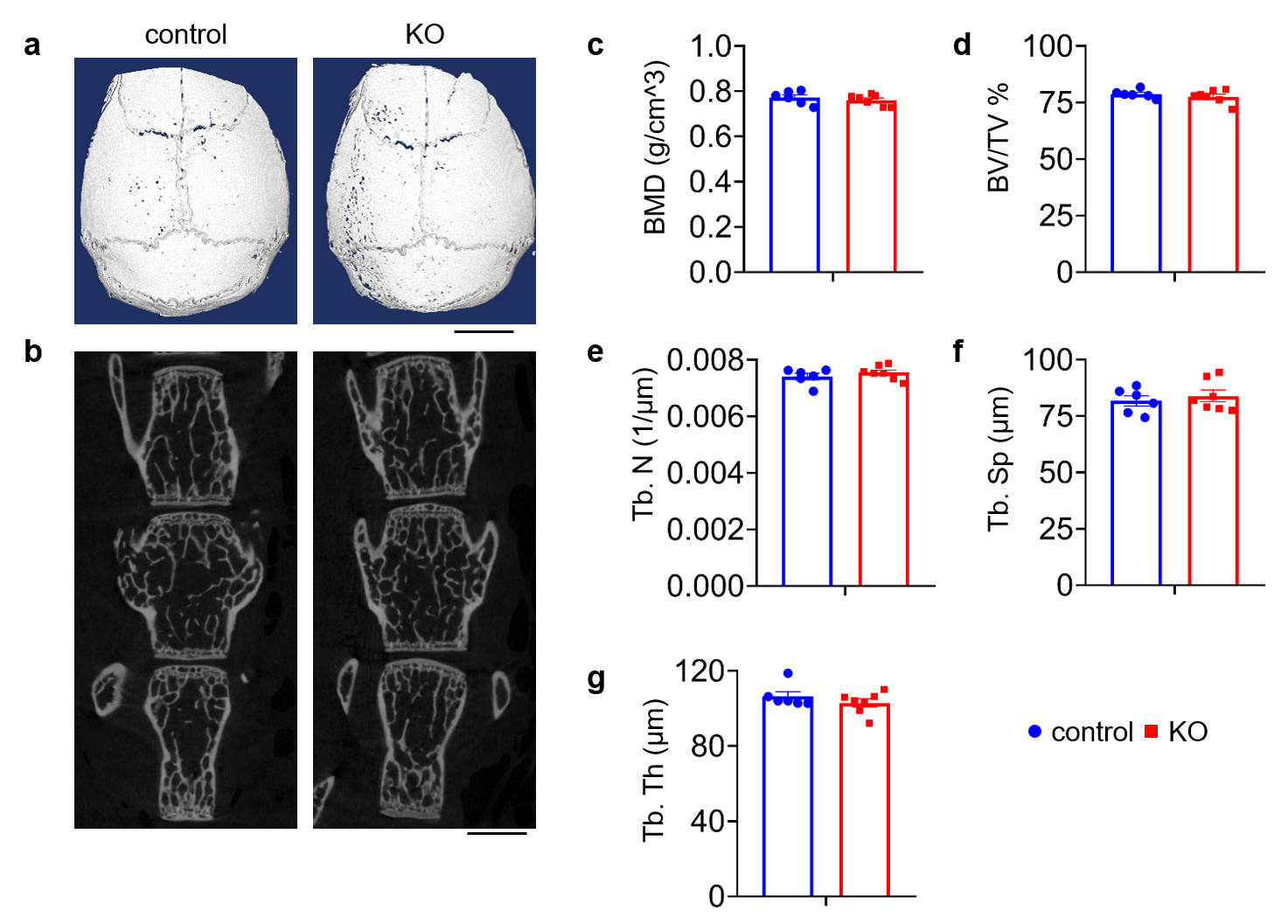


**Supplementary Figure 6. a** Three-dimensional (3D) mico-CT of the calvaria from two groups mice. Scale bar, 500 μm. **b** μCT scans of spinal vertebral bone. Scale bar, 500 μm. **c-g** Quantification of BMD (**c**), BV/TV (**d**), Tb.N (**e**), Tb. Sp (**f**), Tb. Th (**g**) of distal femurs from **a**. *N* = 6 for control female mice and *N* = 7 for KO female mice. Results are showed as mean ± SEM.


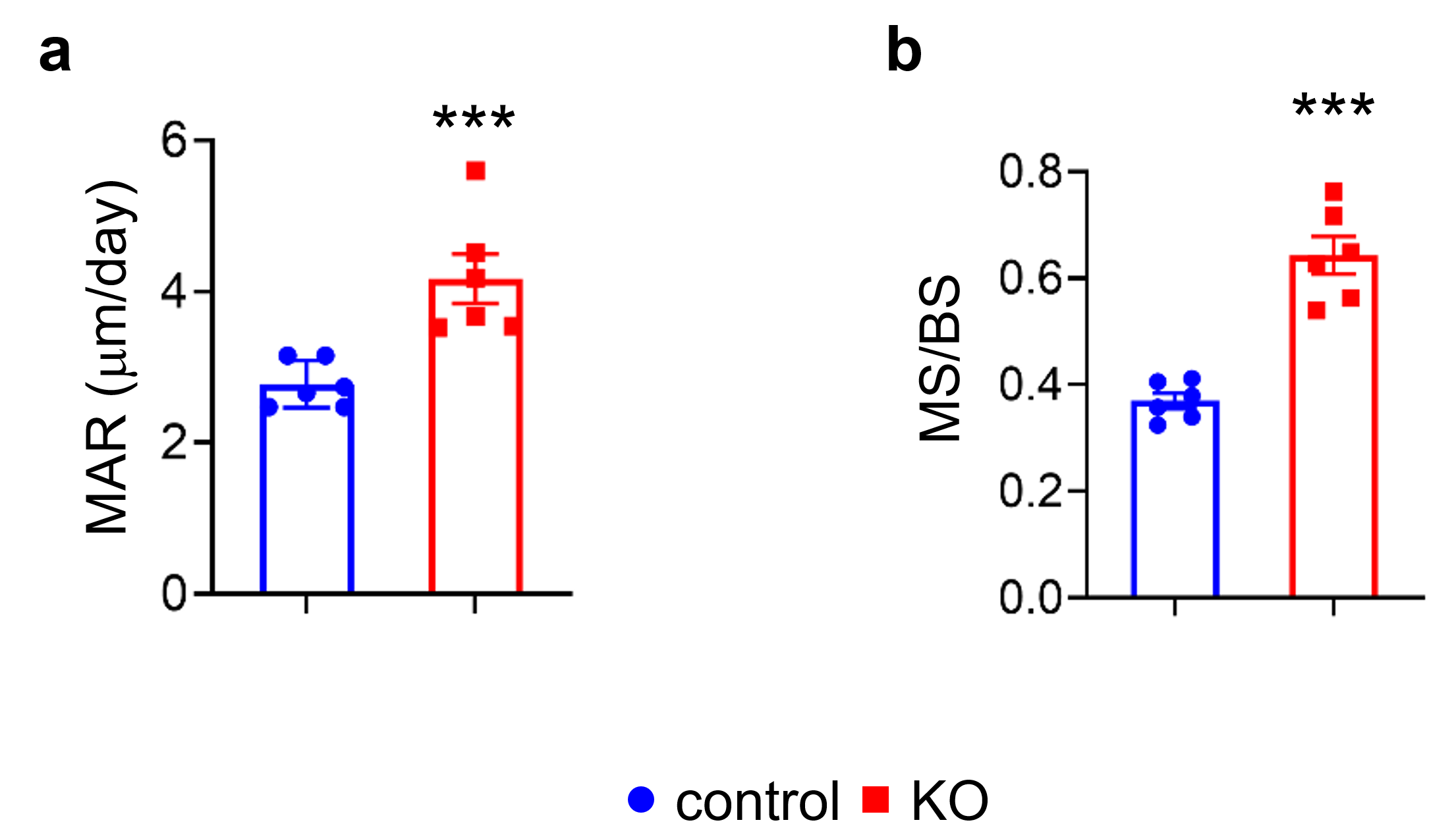


**Supplementary Figure 7. a** Quantification of the mineral apposition rate and **b** mineralizing surface per bone surface of trabecular bone. *N* = 6 for female mice each group. **P* < 0.05, ***P* < 0.01, ****P* < 0.001 vs. controls. Results are showed as mean ± SEM.


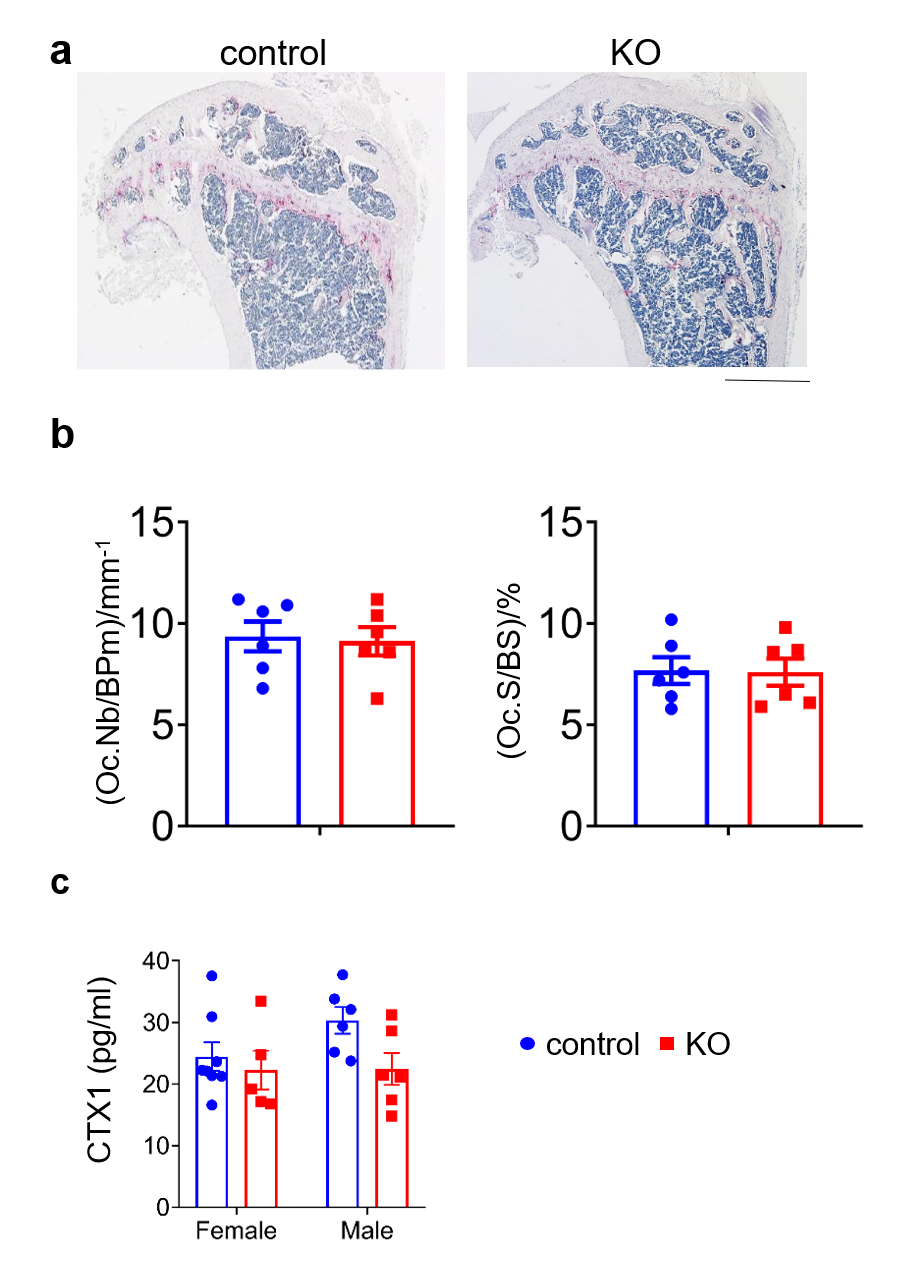


**Supplementary Figure 8. a-b** Tartrate-resistant acid phosphatase (TRAP) staining. Tibial sections from 5-month-old female control and KO mice subjected to TRAP staining. Scale bar, 200 μm. (**a**) Quantification of the osteoclast surface per bone surface (Oc.S/BS) and osteoclast numbers per bone perimeter(Oc.N/BPm). (**b**) *N* = 6 for control, *N* = 6 for KO. **c** Serum levels of collagen type I cross-linked C-telopeptide (CTX-1) from 5-month-old control and KO mice. *N* = 8 for control female mice; *N* = 5 for KO female mice; *N* = 6 for male mice each group. Results are showed as mean ± SEM.


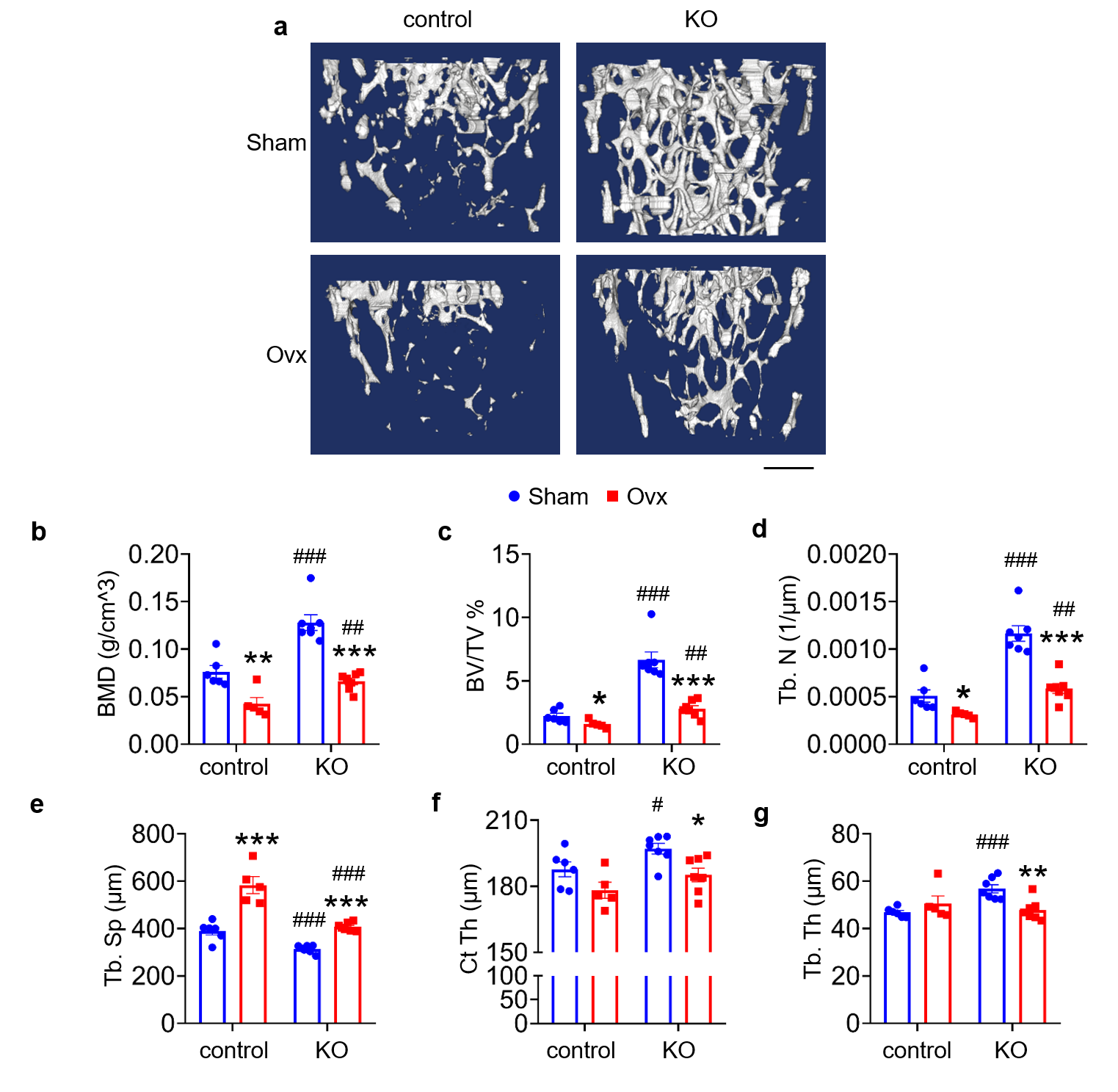


**Supplementary Figure 9. a** Representative μCT images of distal femurs. 4-month-old mice were subjected to sham and OVX operation. Scale bar, 500 μm. **b-g** Quantification of BMD (**b**), BV/TV (**c**), Tb. N (**d**), Tb. Sp (**e**), Ct. Th (**f**), and Tb. Th (**g**) of distal femurs from (**a**). *N* = 6 for control/sham mice; *N* = 5 for control/OVX mice; *N* = 7 for KO/sham mice and *N* = 7 for KO/OVX mice. **P* < 0.05, ***P* < 0.01, ****P* < 0.001, Ovx vs Sham; ^#^*P* < 0.05, ^##^*P* < 0.01, ^###^*P* < 0.001, KO vs control. Results are showed as mean ± SEM.


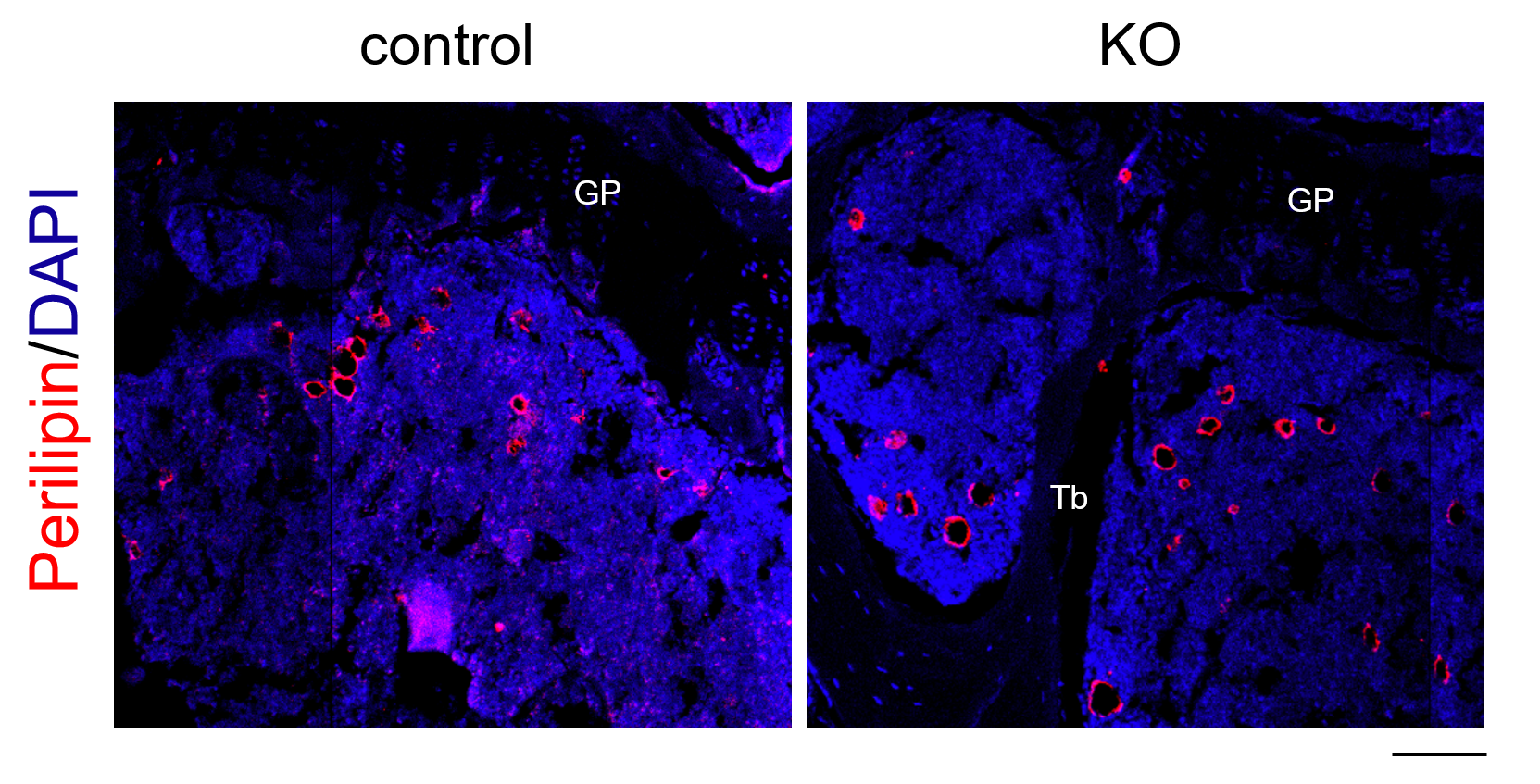


**Supplementary Figure 10.** Perilipin staining. Tibial sections from 5-month-old female mice subjected to IF staining using the Perilipin antibody. Scale bar, 50 μm.


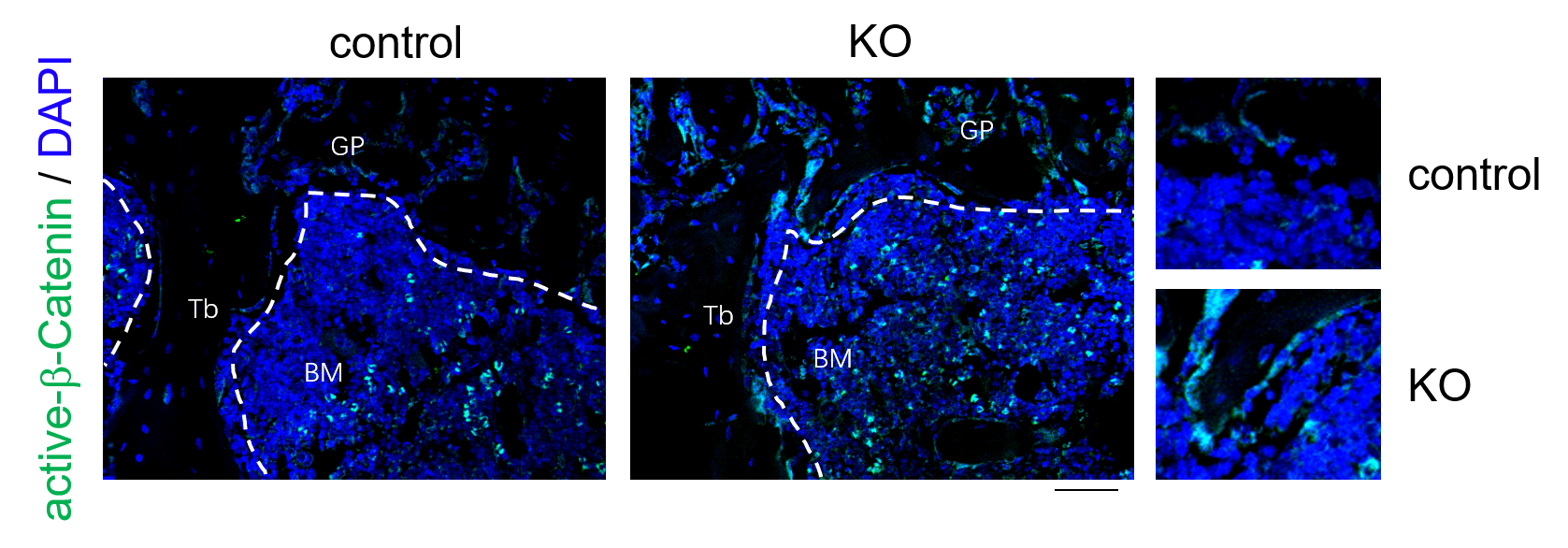


**Supplementary Figure 11.** IF staining of Non-phospho (Active)-β-catenin. Tibial sections from 5-month-old female control and KO mice subjected to IF staining using the Non-phospho (Active)-β-catenin antibody or DAPI. Scale bar, 50 μm.

**Table S1. Antibody information**

| **Name** | **Supplier** | **Cat no.** | **Application/Dilution** |
| --- | --- | --- | --- |
| Sost | Abcam | Ab63097 | WB (1:1000) |
| Gapdh | ZSGB-BIO | TA-08 | WB (1:5000) |
| Runx2 | CST | 12556 | WB (1:5000) |
| Ap2 | Abcam | Ab92501 | WB (1:2000) |
| C/ebpα | Abcam | ab40764 | WB (1:2000) |
| Osx | Abcam | ab22552 | WB (1:2000) |
| Ocn | Bioss | BS-4917R | IF (1:500) |
| Pparγ | CST | 2435 | WB (1:1000) |
| Perilipin | CST | 9349 | IF (1:500) |
| Active β-Catenin | CST | 8814 | IF (1:500) |

**References**

1. Qin, L. *et al.* Osteocyte beta1 integrin loss causes low bone mass and impairs bone mechanotransduction in mice. *J Orthop Translat* **34**, 60-72 (2022).

2. Yan, Q., Gao, H., Yao, Q., Ling, K. & Xiao, G. Loss of phosphatidylinositol-4-phosphate 5-kinase type-1 gamma (Pip5k1c) in mesenchymal stem cells leads to osteopenia by impairing bone remodeling. *J Biol Chem* **298**, 101639 (2022).

3. Gao, H. *et al.* Pinch Loss Ameliorates Obesity, Glucose Intolerance, and Fatty Liver by Modulating Adipocyte Apoptosis in Mice. *Diabetes* **70**, 2492-2505 (2021).

4. Gao, H. *et al.* Kindlin-2 haploinsufficiency protects against fatty liver by targeting Foxo1 in mice. *Nat Commun* **13**, 1025 (2022).

5. Lei, Y. *et al.* LIM domain proteins Pinch1/2 regulate chondrogenesis and bone mass in mice. *Bone Res* **8**, 37 (2020).

6. Xiao, G. *et al.* Critical role of filamin-binding LIM protein 1 (FBLP-1)/migfilin in regulation of bone remodeling. *J Biol Chem* **287**, 21450-21460 (2012).

7. Yu, S. *et al.* Critical role of activating transcription factor 4 in the anabolic actions of parathyroid hormone in bone. *PLoS One* **4**, e7583 (2009).
